# A Web-Based Multidrug-Resistant Organisms Surveillance and Outbreak Detection System with Rule-Based Classification and Clustering

**DOI:** 10.2196/jmir.2056

**Published:** 2012-10-24

**Authors:** Yi-Ju Tseng, Jung-Hsuan Wu, Xiao-Ou Ping, Hui-Chi Lin, Ying-Yu Chen, Rung-Ji Shang, Ming-Yuan Chen, Feipei Lai, Yee-Chun Chen

**Affiliations:** ^1^Graduate Institute of Biomedical Electronics and BioinformaticsNational Taiwan UniversityTaipeiTaiwan; ^2^Department of Electrical EngineeringNational Taiwan UniversityTaipeiTaiwan; ^3^Department of Computer Science and Information EngineeringNational Taiwan UniversityTaipeiTaiwan; ^4^Center for Infection ControlNational Taiwan University HospitalTaipeiTaiwan; ^5^Graduate Institute of Networking and MultimediaNational Taiwan UniversityTaipeiTaiwan; ^6^Information Systems OfficeNational Taiwan University HospitalTaipeiTaiwan; ^7^Department of Internal MedicineNational Taiwan University Hospital and College of MedicineTaipeiTaiwan

**Keywords:** multidrug resistance, surveillance, infection control, information systems, cluster analysis, Web-based services

## Abstract

**Background:**

The emergence and spread of multidrug-resistant organisms (MDROs) are causing a global crisis. Combating antimicrobial resistance requires prevention of transmission of resistant organisms and improved use of antimicrobials.

**Objectives:**

To develop a Web-based information system for automatic integration, analysis, and interpretation of the antimicrobial susceptibility of all clinical isolates that incorporates rule-based classification and cluster analysis of MDROs and implements control chart analysis to facilitate outbreak detection.

**Methods:**

Electronic microbiological data from a 2200-bed teaching hospital in Taiwan were classified according to predefined criteria of MDROs. The numbers of organisms, patients, and incident patients in each MDRO pattern were presented graphically to describe spatial and time information in a Web-based user interface. Hierarchical clustering with 7 upper control limits (UCL) was used to detect suspicious outbreaks. The system’s performance in outbreak detection was evaluated based on vancomycin-resistant enterococcal outbreaks determined by a hospital-wide prospective active surveillance database compiled by infection control personnel.

**Results:**

The optimal UCL for MDRO outbreak detection was the upper 90% confidence interval (CI) using germ criterion with clustering (area under ROC curve (AUC) 0.93, 95% CI 0.91 to 0.95), upper 85% CI using patient criterion (AUC 0.87, 95% CI 0.80 to 0.93), and one standard deviation using incident patient criterion (AUC 0.84, 95% CI 0.75 to 0.92). The performance indicators of each UCL were statistically significantly higher with clustering than those without clustering in germ criterion (*P *< .001), patient criterion (*P *= .04), and incident patient criterion (*P *< .001).

**Conclusion:**

This system automatically identifies MDROs and accurately detects suspicious outbreaks of MDROs based on the antimicrobial susceptibility of all clinical isolates.

## Introduction

During the last few decades, the nonspecific nature and overlapping spectra of early phase infection caused by bacteria and other pathogens have resulted in the overuse of antimicrobials [1]. This parallels a relentless increase in the number and types of microorganisms resistant to these medicines. Patients infected with resistant organisms are more likely to receive inappropriate initial therapy and are, thus, associated with higher mortality, morbidity, and medical costs [2-3]. The emergence and spread of multidrug-resistant organisms (MDROs) are considered to be causing a global crisis [1,4]. To resolve this problem, the WHO has made antimicrobial resistance an organization-wide priority and the focus of the 2011 World Health Day.

Patients can become infected, or a carrier, by exposure to an MDRO-contaminated environment (including medical devices), close contact with a carrier, or following the use of antimicrobial agents. Multidrug-resistant organisms may spread further, resulting in outbreaks and compromising patient safety [5-10]. Combating antimicrobial resistance requires prevention of transmission of resistant organisms and improved use of antimicrobials [1]. The core components of infection control strategies for MDRO, therefore, include early identification, monitoring, and prevention of spread [2]. One or more hospital staff, usually infection control personnel, is responsible for identifying and tracking down carriers or patients with infections caused by epidemiologically important MDROs and preventing further spread. This is usually conducted by reviewing the laboratory reports of all clinical isolates, on a daily or weekly basis, to recognize any unusual clustering or increases in the numbers of specific bacteria or by identifying antimicrobial susceptibility testing results (antibiogram) at indicated units within a period. The key information from MDRO surveillance consists of the species of MDRO, antibiogram, space (unit), and time. The identification of potential MDRO outbreak is a complex process and, therefore, extremely challenging for infection control personnel at a large teaching hospital.

Information technology is expected to improve efficiency in automated surveillance and infection control [11-13]. Previous studies have devised and implemented computer-assisted infection control surveillance or outbreak detection systems, and these have proved beneficial in MDRO surveillance [14-17]. However, prior attempts to establish a Web-based MDRO surveillance system that allows automated real-time integration, analysis, and interpretation have been few. In such a system, visualization methods are also important for the clear presentation of the proximity of time and space, and the species of MDRO, and to facilitate data-driven decision-making.

The present study developed a Web-based MDRO surveillance and outbreak detection information system at a teaching hospital in Taiwan. The system adheres to a Service-Oriented Architecture (SOA) and to Health Level Seven (HL7). It incorporates rule-based classification and cluster analysis of all reported antibiogram profiles, implements control charts with surveillance rules and hierarchical clustering for data analysis, and provides useful information to facilitate the timely targeting of the correct unit by infection control personnel for appropriate intervention. The study includes evaluation of the system performance.

## Methods

### Hospital Setting and Infection Control Program

National Taiwan University Hospital (NTUH), a 2200-bed major teaching hospital in Taiwan, provides both primary and tertiary medical care. In 2010, it served 87, 559 inpatients and 2 ,181, 764 outpatients and received 106, 090 emergency visits. A total of 248, 362 specimens were sent for bacterial isolation and identification. There were 20,472 MDROs, and other surveillance target organisms (described in Appendix 1) were identified.

Prospective, hospital-wide on-site surveillance of health care-associated infection was conducted, from its initiation in 1981, by way of weekly visits by infection control personnel to all inpatient units [18]. In addition, infection control personnel monitored culture results from the clinical microbiology laboratory, on a daily basis, to identify any clustering of epidemiologically important MDROs. Oral reminders and formal feedback were provided to the hospital units to strengthen infection control measures. Site visits, audits, and investigations were conducted periodically and if necessary.

A Web-based MDRO surveillance system was developed to automatically and instantaneously detect and monitor the hospitalized patients with MDRO carriage. It has been executed routinely since October 2010. The following sections describe the system architecture and software components. The performance of the system was also evaluated. System Architecture

**Figure 1:**
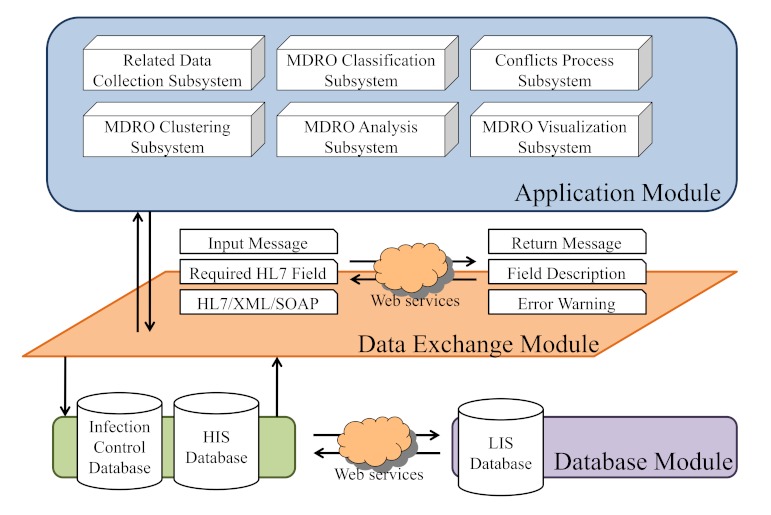
The system architecture of the Web-based multidrug-resistant organisms (MDRO) surveillance system.

The MDRO surveillance system includes an application module, a data exchange module, and a database module (Figure 1). The database module consists of the infection control database, health information system (HIS) database, and laboratory information system (LIS) database. The data exchange module connects the application services to the database services through an SOA (service oriented architecture). The HL7-embedded Extensible Markup Language (XML) formatted data are implemented in the data exchange module [19-20] and support message management, routing, mapping, and database access. The application module consists of software components. The MDRO surveillance services can easily be provided to other heterogeneous information systems because of their adherence to SOA and HL7 standards.

### Software components

**Figure 2:**
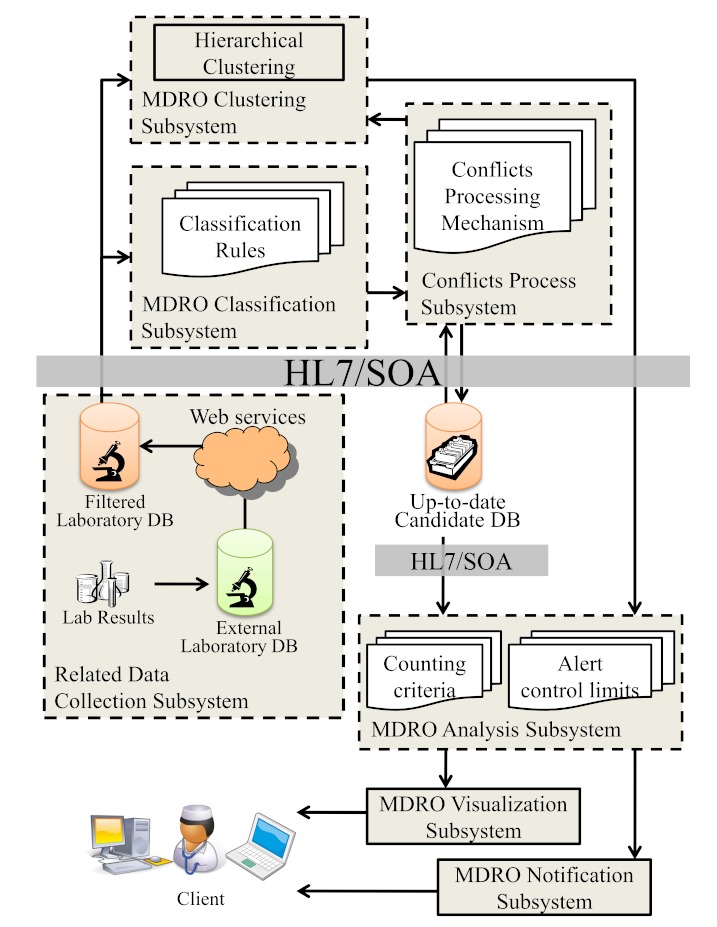
The software components of the application module consist of 7 subsystems.

The software components of the application module consist of 7 subsystems for data collection, conflict processing, MDRO classification and clustering, analysis, visualization, and notification (Figure 2). Data from the clinical microbiology laboratory are collected by Web service from the LIS, mapped, and classified according to the predefined criteria of MDRO and other surveillance target organisms (Appendix 1). Multidrug-resistant organisms are stored in the up-to-date candidate database after processing of the conflicts between preliminary and final reports. Meanwhile, the clinical microbiology laboratory data in the filtered laboratory database are grouped by cluster analysis. The MDRO candidates are then analyzed by counting criteria and alert upper control limits (UCLs). The results of analysis are displayed in a Web-based user interface. This Web-based MDRO surveillance system monitors MDROs on a daily basis, and every hour if indicated, and is in conjunction with the HIS, which offers a single entry point to the Web-based interface by way of a browser. The following subsections detail each subsystem. Components of the system, including the related data collection, MDRO classification, conflict process, and notification subsystem have been described previously [21] (Appendix 2). The target organisms for surveillance and definition of the multidrug-resistant organisms are shown in Appendix 1, classified into 5 categories according to classification logics.

### MDRO Clustering Subsystem

The MDRO clustering subsystem automatically compares and analyzes the antimicrobial susceptibility testing results among organisms. For each organism against an antimicrobial agent, there are 4 possible results: R (resistant), I (intermediate), S (sensitive), and missing (when no data are available). For example, ten antimicrobial agents were evaluated for one organism: AN, ATM, CAZ, CIP, FEP, GM, LVX, MEM, SAM, and TIM. The results were I, R, R, I, R, R, S, S, S, and S, respectively. The result was considered an ordered sequence, IRRIRRSSSS, which was analyzed, and the similarities were calculated by hierarchical clustering. Different from K-means clustering, hierarchical clustering does not require determining the number of clusters, and clusters are separated based on their distance [22]. More importantly, the distance concept of hierarchical clustering meets the clinical meaning of organism clustering, and infection control personnel could easily understand the degree of distance between organism clusters.

**Figure 3:**
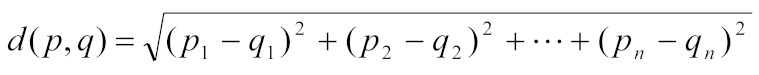
Equation 1.

First, Euclidean distance between each organism was calculated. The distance between two 1-by-n vectors x_p _and x_q _in one dimension was the absolute value of the difference between their coordinates (defined as in Figure 3). The result of this equation is commonly known as a distance matrix.

**Figure 4:**
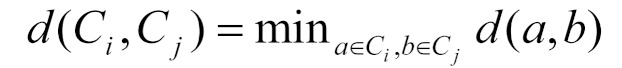
Equation 2.

The organisms were then grouped into a binary hierarchical cluster tree, which is a multilevel hierarchy, where clusters at one level are joined as clusters in the following level [23]. In this step, pairs of objects that were in proximity were linked using the single linkage. The single linkage uses the smallest distance generated in the previous step between different objects and produces long clusters with large diameters [22,24]. That is, two clusters were merged according to the minimum distance, and it was the property that was needed in this research to cluster each organism under less strict rules (Figure 4).

Following the pairing of objects into binary clusters, the newly formed clusters were grouped into larger clusters until a hierarchical tree was formed, thus using agglomerative methods. Agglomerative hierarchical clustering is a bottom-up clustering method that has been studied and used extensively [25]. In the infection control personnel’s view, this bottom-up strategy is more similar to the concept of organism clustering than divisive (top-down) strategy.

**Figure 5:**
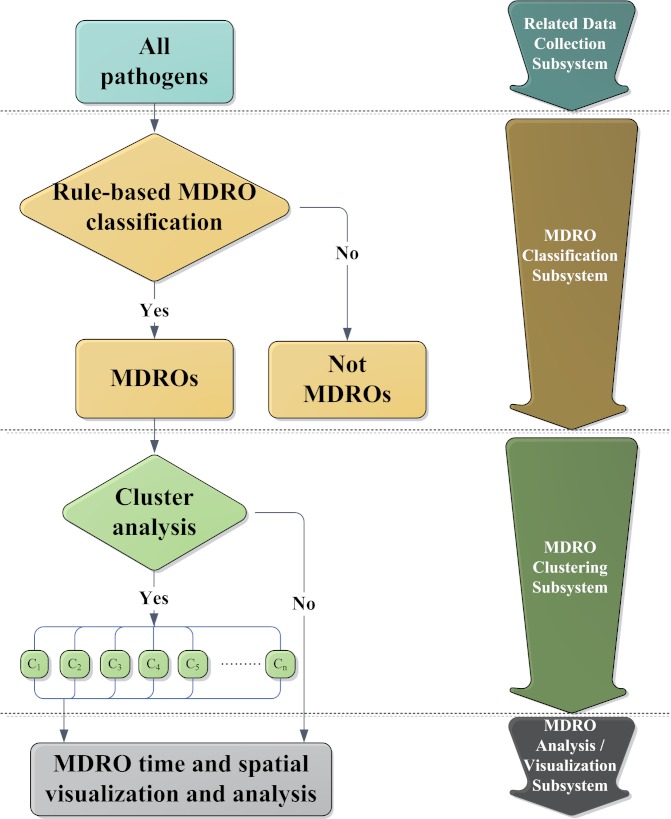
Flowchart of MDRO cluster analysis.

The clustering flowchart is shown in Figure 5. With clustering, the MDROs of each class from the rule-based MDRO classification subsystem were clustered in the MDRO clustering subsystem by R [23,26]. The components of a cluster changed by altering the cutting Euclidean distance: the smaller the cutting distance used, the more clusters that were obtained. Different clusters were analyzed separately in the MDRO analysis subsystem. Without clustering, the MDROs of each class were analyzed directly after MDRO classification.

### MDRO Analysis Subsystem

To assist infection control personnel with judgment and decision making, the MDRO candidates were analyzed based on the temporal and spatial distribution of patients with MDRO colonization or infection. The system used three counting criteria: germ criterion, patient criterion, and incident patient criterion (Table 1). For the germ criterion, the system counted the numbers of positive results in MDRO culture reports in a given period (in this system, 1 week). One patient may have one or more reports (from different body sites or the same body sites at different times). For the patient criterion, the system counted the numbers of patients who have MDRO specimen reports in a given period. When a patient had more than one MDRO specimen report (eg, MRSA isolated from a blood sample, the tip of the central venous catheter, and pus from a bed sore) in a given period, they were counted only once. The data, thus, present the disease burden at a given time. For the incident patient criterion, this system counted the numbers of patients who were newly colonized or infected by an MDRO if they did not have MDRO culture reports in the 30 days previous to the release of the current MDRO culture report. The data were approximately, although not equal to, the number of patients with newly acquired MDRO during their hospital stay.

**Table 1:** The definition and rationale of three counting criteria for multidrug-resistant organism surveillance and outbreak detection system.

Criteria	Definition	Rationale
Germ criterion	The numbers of positive results in MDRO culture reports.	More MDROs isolated from an individual or a group of patients may represent the higher probability of spreading.
Patient criterion	The numbers of patients who have MDRO specimen reports.	The data present the disease burden at a given time. This data may help for resource allocation, such as the use of single room isolation versus cohorting of more than one patients with the same MDRO colonization/infection in the same room.
Incident patient criterion	The numbers of patients who were newly colonized or infected by MDRO if they did not have MDRO culture reports in the 30 days previous to the release of the current MDRO culture report.	The number of patients with newly acquired MDRO during their hospital stay. The higher incident of patients represents the poorer performance of infection control practice. More infection control personnel are likely required to remind, audit, or practice another intervention.

This system also included alert UCLs to identify suspicious outbreaks:

*n *SD: With this UCL, an alert is defined as *n *standard deviations (*n *SD) above the mean (central line).

*m*% CI: With this UCL, an alert is defined as lying outside of the *m*% confidence interval (CI) [27]. The value of *m*, defined as the confidence coefficient, could be assigned by users.

These alert UCLs were calculated based on a defined observation period in the past. For example, if the surveillance month was December 2008 and the defined observation period was 1 year, these UCLs were calculated based on the data from December 2007 to November 2008. Due to 3 SD UCL being stricter than 2 SD and 1 SD UCL, there are fewer events flagged as outbreaks by 3 SD UCL. It is anticipated that events that were flagged outside of 2 SD include those defined by 3 SD. Events that were flagged outside of the 95% confidence interval include the 99% confidence interval. The optimal UCL will be determined by the impact (severity or chance for control) of the incident event or the disease surveyed. That is, the more severe the outcome, the more that the lower UCL is preferred.

### MDRO Visualization Subsystem

**Figure 6:**
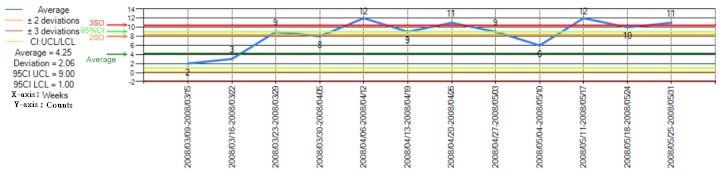
Control chart for visualization of the time series of the MDRO classification results in a defined patient population (eg, the whole hospital). This chart displays the numbers of vancomycin-resistant Enterococcus faecium isolates (germ criteria, y-axis) by week from March 9, 2008, to May 31, 2008 (x-axis).

**Figure 7:**
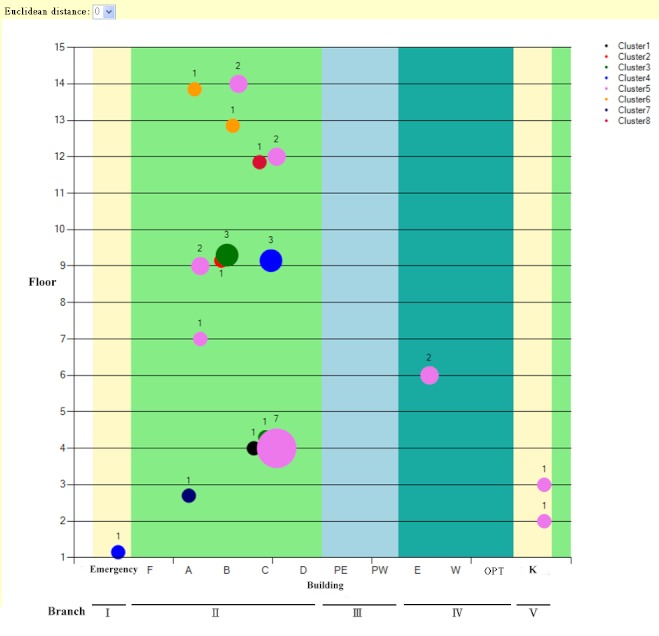
Bubble chart for visualization of the spatial distribution of MDRO clustering results. This chart display the spatial distribution of vancomycin-resistant Enterococcus faecium isolated from April 6, 2008, to April 26, 2008, and clustered with Euclidean distance equal to zero and single linkage.

After the MDRO analysis process, a line chart was used to describe time trends of the MDRO count (Figure 6). For the outliers, the clustering results were further presented in a bubble chart to illustrate the spatial distribution of the MDROs, the similarities of the antimicrobial susceptibility testing results, and the MDRO counts in different areas (Figure 7).

**Figure 8:**
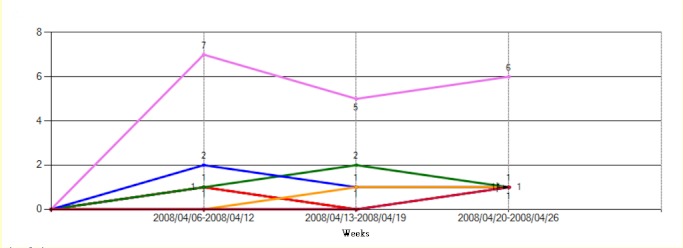
Line chart for visualization of the time trend of MDRO clustering results. This chart display the number of clustered vancomycin-resistant Enterococcus faecium isolates (germ criteria, y-axis), from April 6, 2008, to April 26, 2008, with Euclidean distance equal to zero and single linkage.

The bubble chart shown in Figure 7 represents the clustering results with spatial distribution. The x-axis represents the branch or building of the ward where the patient with MDRO stayed; the y-axis of the bubble chart represents the floor of the ward. The bubble size represents the number of MDROs in a specific ward, which is defined by the x and y axes. The different colors of bubbles indicate that the MRDOs belonged to different clusters. The line chart, describing the time trends of clustering results, supplements the bubble chart (Figure 8).

In addition to time trends and spatial distribution of numbers of MDROs, medical staff can also retrieve detailed information on specimen reports that interest them. With the 2-level embedded function, they can rapidly respond to control the further spread of MDROs. This system facilitates the stream processing of the occurrence of MDROs in the population levels, as well as the identification of patients with MDRO carriage and those in need of special attention.

### Evaluation of Performance

Two levels of performance evaluation were conducted on the Web-based MDRO surveillance system: MDRO detection and classification of specimen reports, and outbreak detection. The performance of the first part of the system described previously [21] (Appendix 2), includes the time cost and accuracy of MDRO detection and classification of specimen reports, and the proportion of patients receiving contact isolation. The proportion of contact isolation orders was defined according to the number of patients with contact isolation orders per 100 patients with MDRO specimen reports. These indicators were compared in the absence of the Web-based MDRO surveillance system (from April 1, 2008, to July 9, 2008) and in the presence of the system (from September 1, 2008, to December 9, 2008). The time cost in the absence of the MDRO system was the average person-minute to identify the MDRO among a set of 100 clinical isolates using antimicrobial susceptibility testing results by 10 hospital staff, multiplied by the total number of clinical isolates per day, and divided by 100.

**Figure 9:**
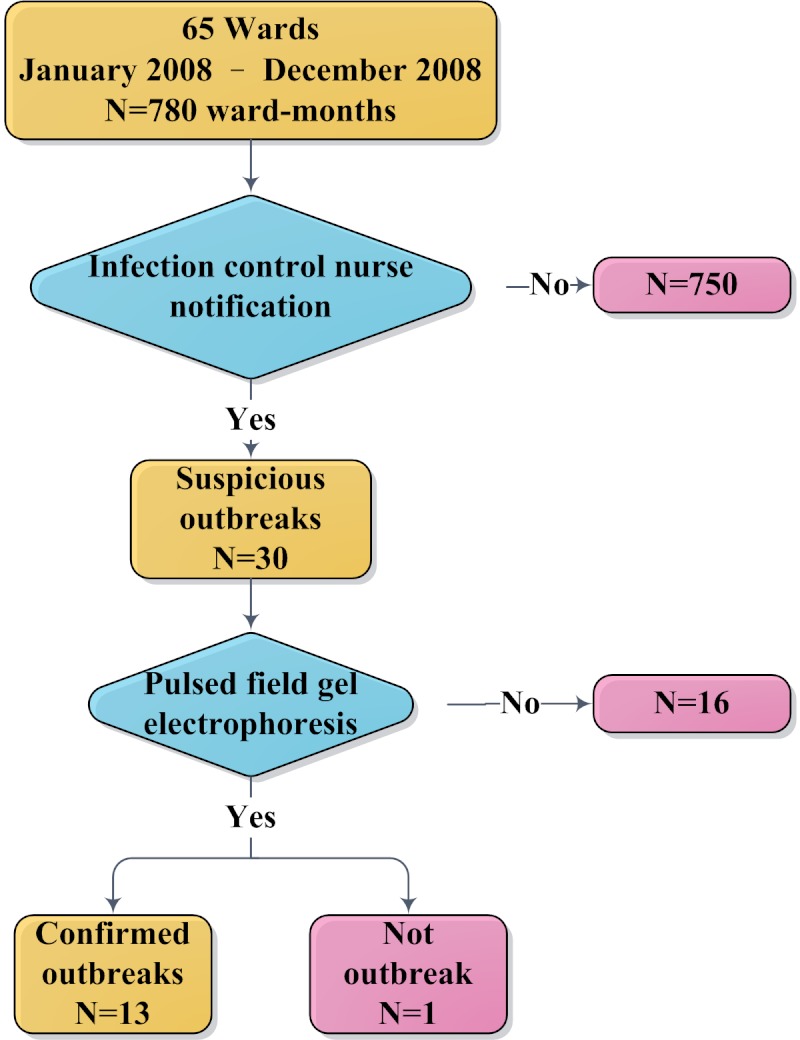
Data description of suspicious and confirmed outbreaks due to vancomycin-resistant Enterococcus.

**Figure 10:**
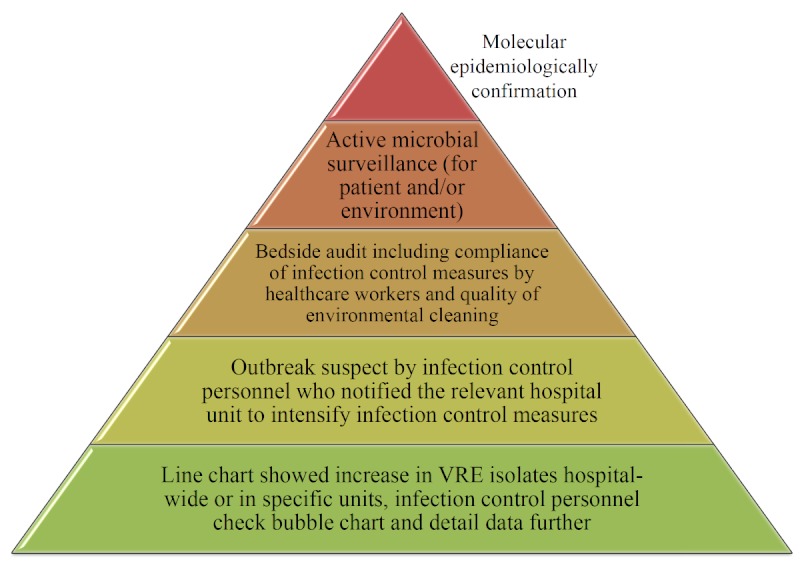
The pyramid of vancomycin-resistant Enterococcus outbreaks identified, investigated, and confirmed, showing the concept of the integrated Web-based surveillance and outbreak detection system and infection control personnel expertise.

The MDRO outbreak detection with clustering has been available since April 1, 2011. According to the hospital-wide prospective infection control surveillance data, vancomycin-resistant *Enterococcus *species (VRE) were the leading pathogens to cause outbreaks in 2008. Thus, we used the prospectively defined VRE outbreak data in 2008 (before the implementation of the system) to evaluate the MDRO outbreak detection performance (Figure 9). A suspicious outbreak was defined when a group of patients displayed temporal and spatial clustering of isolations of VRE with identical antibiogram from clinical specimens, and infection control personnel notified the relevant hospital unit to intensify infection control precautions. Only a number of the outbreaks, usually those persisting despite intervention, were investigated further including active microbial surveillance and confirmed by pulsed field gel electrophoresis (PFGE) (Figure 10). A confirmed outbreak was defined by the presence of a predominant clone of VRE from clinical specimens as determined by PFGE among a group of patients with temporal and spatial clustering. The details of the outbreak investigation and the infection control program for VRE were described previously [28]. 

The stability of the hierarchical clustering algorithm on this dataset was ascertained by clValid R-package and used for comparing different cutting distances of the tree [29]. The package was often used for biomedical clustering [30-32], providing stability measures, including average proportion of non-overlap (APN), average distance (AD), average distance between means (ADM), and figure of merit (FOM). The definitions of these measures were described in previous research [29]. These stability measures compare the results from clustering based on the full data to clustering based on removing each column one at a time.

To detect a suspicious outbreak, sensitivity, specificity, positive predictive value (PPV), negative predictive value (NPV), area under the receiver operating characteristic curve (AUC), and CI of AUC were used to select an optimal UCL for predicting MDRO suspicious outbreaks [33]. The suspicious outbreaks of VRE were identified by 7 UCLs, including the upper 99% CI, upper 95% CI, upper 90% CI, upper 85% UCL, 3 SD, 2 SD, and 1 SD. The UCLs were generated based on data from the previous year.

## Results

The time cost of MDRO detection and classification of specimen reports in the absence of the Web-based MDRO surveillance system was 450 **± **192.2 min per day; in the presence of the system it was 0 min (*P *< .001). That is, implementation of this system may save approximately 1 person-day of the 10 infection control personnel daily. The accuracy of MDRO detection was 63.9 **± **26.4% by infection control personnel; the system was 100% (*P *< .001). The proportion of contact precautions of incident patients increased after implementation of the system (16.5 **± **16.5% versus 25.5 **± **22.1%, *P *= .001) [21] (Appendix 2).

The system evaluated all specimens sent to the routine bacteriology laboratory collected from patients hospitalized in 65 wards during a 12-month study period in 2008. Data from each ward were analyzed by month. Among 780 ward-months evaluated, there were 30 suspicious outbreaks identified by infection control nurses. Of 14 suspicious outbreaks, PFGE study of the VRE isolates confirmed 13 as outbreaks (Figure 9).

Tables 2 to 4 display the system’s performance for VRE outbreak detection according to defined UCLs using the germ criterion, patient criterion, or incident patient criterion, with and without clustering. The optimal UCL was upper 90% CI using the germ criterion (number of VRE isolates) with clustering (AUC 0.93, 95% CI 0.91 to 0.95), followed by upper 85% CI using the patient criterion (number of patients with VRE isolated from clinical specimens) (AUC 0.87, 95% CI 0.80 to 0.93), and 1 SD using the incident patient criterion (number of patients with VRE not identified in previous months) (AUC 0.84, 95% CI 0.75 to 0.92). Appendices 3 to 5 display the details of the system’s performance for VRE outbreak detection according to each criterion, with and without clustering. The performance indicators of each UCL were statistically significantly higher with cluster analysis than those without cluster analysis in germ criterion (*P *< .001), patient criterion (*P *= .04), and incident patient criterion (*P *< .001).

The stability of clustering is shown in Table 5. AD and FOM measures were optimized in all criteria with cutting Euclidean distance being zero.

**Table 2:** Performance in outbreak detection according to germ criterion and a upper control limit defined by 90% confidence interval, with and without clustering.

Parameter	Without clustering	With clustering (d=0)^g^
Sensitivity (%)^a^	90.0 (27/30)	100 (30/30)
Specificity (%)^b^	84.0 (630/750)	86.7 (650/750)
PPV (%)^c^	18.4 (27/147)	23.1 (30/130)
NPV (%)^d^	99.5 (630/633)	100 (650/650)
AUC^e ^(95% CI^f^)	0.87 (0.81-0.94)	0.93 (0.91-0.95)

^a ^Sensitivity = TP/(TP+FN) (where TP (true positive) is an outbreak correctly identified as an outbreak, and FN (false negative) is an outbreak wrongly identified as a non-outbreak).

^b ^Specificity = TN/(TN+FP) (where TN (true negative) is a non-outbreak correctly identified as a non-outbreak, and FP (false positive) is a non-outbreak wrongly identified as an outbreak).

^c ^Positive predictive value (PPV) = TP/(TP+FP) (variables defined above).

^d ^Negative predictive value (NPV) = TN/(TN+FN) (variables defined above).

^e ^AUC: area under receiver operating characteristic curve.

^f ^CI: confidence interval.

^g ^d: cutting Euclidean distance.

**Table 3:** Performance in outbreak detection according to patient criterion and a variety of upper control limits defined by 1 standard deviation and 85% confidence interval, with and without clustering.

	Without clustering	With clustering (d=1)^g^	With clustering (d=0)		
	1 standard deviation	1 standard deviation	Upper 85% CI		
Sensitivity (%)^a^	86.7 (26/30)	86.7 (26/30)	90.0 (27/30)		
Specificity (%)^b^	83.5 (626/750)	83.2 (624/750)	83.6 (627/750)		
PPV (%)^c^	17.3 (26/150)	17.1 (26/152)	18.0 (27/150)		
NPV (%)^d^	99.4 (626/630)	99.4 (624/628)	99.5 (627/630)		
AUC^e ^(95% CI^f^)	0.85 (0.78-0.92)	0.85 (0.78-0.92)	0.87 (0.80-0.93)		

^a-g ^as shown in Table 2 footnotes.

**Table 4:** Performance in outbreak detection according to incident patient criterion and a variety of upper control limits defined by 1 standard deviation and 90% confidence interval, with and without clustering.

	Without clustering	With clustering (d=1)^g^	With clustering (d=0)
	Upper 90% CI	Upper 90% CI	1 standard deviation
Sensitivity (%)^a^	76.7 (23/30)	76.7 (23/30)	80.0 (24/30)
Specificity (%)^b^	83.9 (629/750)	83.9 (629/750)	87.3 (655/750)
PPV (%)^c^	16.0 (23/144)	16.0 (23/144)	20.2 (24/119)
NPV (%)^d^	99.0 (629/636)	99.0 (629/636)	99.1 (655/661)
AUC^e ^(95% CI^f^)	0.80 (0.72-0.89)	0.80 (0.72-0.89)	0.84 (0.75-0.92)

^a-g ^as shown in Table 2 footnotes.

**Table 5:** Stability of clustering with germ, patient, and incident patient criteria and a variety of cutting Euclidean distance.

Criteria	Cutting distance^a^	APN**^b^**	AD**^c^**	ADM**^d^**	FOM**^e^**
Germ criterion	0	0.00	0.12	0.12	0.18
Patient criterion	0	0.00	0.12	0.12	0.17
	1	0.00	0.78	0.01	0.20
Incident patient criterion	0	0.00	0.13	0.13	0.18
	1	0.00	0.83	0.01	0.21

^a ^d: cutting Euclidean distance.

^b ^Average proportion of non-overlap (APN).

^c ^Average distance (AD).

^d ^Average distance between means (ADM).

^e ^Figure of merit (FOM).

## Discussion

### Principal Results

This study demonstrated the concept that a Web-based MDRO surveillance and outbreak detection information system provides useful information to facilitate the timely targeting of the correct unit by infection control personnel for appropriate intervention and described how to achieve a fine balance in the use of automated cluster detection tools between capturing all statistical clusters versus capturing all clinically meaningful clusters (Figure 10).

This study established a variety of UCLs and clustering methods for the detection and intervention of outbreaks caused by MDROs. The highest area under the ROC curve for detecting VRE outbreaks was 0.93 using a UCL defined by 90% CI with clustering and the germ criterion. In all criteria, the performance indicators of each UCL were statistically significant higher with clustering than those without clustering. For the stability of clustering, although ADM measure was optimized in all criteria with cutting Euclidean distance being 1, AD and FOM measures were optimized in all criteria with cutting Euclidean distance being zero. Average distance (AD) and FOM measures compute distance and variance of observations in the same cluster between original data and single column removed data. Because the observations in a cluster have the same property with cutting Euclidean distance being zero, the effect of removing a single column in AD and FOM measures could be smaller than others. Different from that, ADM computes the distance of the cluster center for observations placed in the same cluster between the original data and single column removed data. The distance between cluster centers could increase as the number of clusters increases. Even though ADM was not optimized with cutting Euclidean distance being zero, it was a relatively small value in the range from zero to infinity.

The optimal UCLs for detecting outbreaks of VRE in 2008 were presented; however, whether these UCLs remain useful in different MDROs warrants further study. Therefore, this system designated confidence coefficients or multiples of standard deviation to provide the different UCLs, within a user-friendly interface, and incorporates a greater number of possible future scenarios. Infection control experts can, therefore, select 1 or more of these integrated UCLs, which are useful in specific scenarios, then manage the suspicious outbreak with minimal delay. In addition, prospectively defined VRE outbreak was recorded by month; therefore, the performance of the system was determined by month. The control chart presents the number of VRE isolates or patients by week. The system could, therefore, provide earlier detection of suspicious outbreak than detection by infection control nurses.

The traditional expert-defined MDRO classification system suffers from blind spots: it can fail to notice undefined new organisms. It classifies MDROs according to expert-defined rules; therefore, if organisms are not within the defined classification rules, they escape detection. To solve this problem, this study’s new Web-based MDRO-surveillance system combines cluster analysis and traditional MDRO classification. Unlike the traditional MDRO classification system, it groups MDROs according to the antimicrobial susceptibility profiles in cluster analysis. If the unexpected MDROs have antibiogram profiles, the cluster analysis, therefore, also analyzes their suspicious outbreak.

In accordance with the results of previous studies, including those of Kho et al [14], using a computer-assisted system provided a higher proportion of contact isolations than without computer assistance. A computerized decision support system could further improve risk measurement in other aspects of health care [34-35]. The present computer-assisted MDRO surveillance system can provide precise and consistent support to the health care provider [14,16,36-37] and demonstrates usefulness for infection control. Huang et al implemented an automated statistical software that provided valuable real-time guidance by both identifying otherwise unrecognized outbreaks and preventing the unnecessary implementation of resource-intensive infection control measures that interfere with regular patient care [38]. Different from that, this system is Web-based and can be easily adapted by others because of adherence to SOA and HL7 standards. The SOA can also be seamlessly integrated into the HIS and facilitate infection control personnel in obtaining more information from patients. Furthermore, the Web-based system is not only integrated with a clustering algorithm but also provides a visualization tool and adjustable surveillance parameters within a user-friendly interface for MDRO surveillance.

### Limitations

Although our results suggested that the Web-based MDRO surveillance system performs well, it did have several limitations. First, the affected factors of the system include data integrity and instantaneity. The system bases its MDRO classifications and clustering on antibiogram profiles of organisms; thus, it neglects organisms without antimicrobial susceptibility testing results. Second, the performance of the outbreak detection system was evaluated based on the suspicious outbreaks, but not those with PFGE confirmation. Pulsed field gel electrophoresis is currently considered the gold standard for molecular epidemiological characterization of VRE outbreaks [28,39]. However, only some of the outbreaks, usually those persisting despite intervention, were investigated further and confirmed by PFGE (Figure 10). Thus, if limited to those with PFGE, confirmation might lead to underestimation of the occurrence of outbreak.

Third, low PPV risks alert fatigue, particularly if too many alerts are deemed clinically insignificant by infection control personnel, is an important issue. Nevertheless, the important contribution of this system was to provide a user-defined, adoptable, and flexible tool, and to facilitate and assist MDRO surveillance. Fourth, this system has yet to incorporate appropriate statistical analysis for rare events. The rare events in historical data, which defined the central line and UCLs and were used to provide a baseline, were both zero. In 2007, VRE were very rarely isolated from the hospitalized patients (historical data used to define the UCLs). This system is, therefore, always sensitive to new cases, which increases the false positive rate. Fifth, the system did not active alert for any outbreak; all UCL selected outbreaks were only flagged in the line chart and bubble chart waiting for infection control personnel to check, judge, and act accordingly.

Sixth, the contribution of the Web-based surveillance system to any decrease in the rate of incident MDROs detected or the rates of health care-associated infections was unclear. The rate of VRE colonization/infection at this hospital was less than predicted after 2009 [40]. However, hand hygiene promotion and active surveillance also contributed. Finally, the indicators that were used for evaluating MDRO detection and classification of specimen reports were compared over different time periods, which limited the strength of the evidence for the system. Ideally randomization would be used, over the same time period comparing the performance between absence and presence of system, but this isn’t always possible or feasible. Similar evaluation methods had been used in previous studies [41-43].

### Conclusion

In conclusion, this study presents a Web-based MDRO surveillance system that automatically and efficiently classifies, and accurately clusters, MDROs according to the antimicrobial susceptibility profiles, thus detecting potential MDRO clustering. The data connections optimally represent the full conceptual content of the data, allowing automated integration and data-driven decision making. The results may alert hospital personnel to implement contact precaution measures for patients with MDROs. The system is able to save 1 person-day for 10 infection control nurses and also to instigate outbreak intervention and management. As of May 15, 2012, the system was still used for MDRO surveillance and has become an indispensable tool for infection control personnel’s daily work. Implementing this system could, therefore, improve patient safety as well as the quality of medical care in a hospital.

## Multimedia Appendix 1

Target organisms for surveillance and definition of the multidrug-resistant organisms. The patterns are classified into 5 categories according to classification logics.

## Multimedia Appendix 2

Real-time Automated MDRO Surveillance System (Reference 21).

## Multimedia Appendix 3

Performance in outbreak detection according to a variety of germ criterion and upper control limits (UCL), with and without clustering analysis.

## Multimedia Appendix 4

Performance in outbreak detection according to patient criterion and a variety of upper control limits (UCL), with and without clustering analysis.

## Multimedia Appendix 5

Performance in outbreak detection according to incident patient criterion and a variety of upper control limit (UCL), with and without clustering analysis.

## Abbreviations

AD: Average distance
ADM: Average distance between means
APN: Average proportion of non-overlap
AUC: area under the receiver operating characteristic curve
CI: confidence interval
DB: database
FOM: Figure of merit
HIS: health information system
HL7: Health Level Seven
LIS: laboratory information system
MDRO: multidrug-resistant organisms
MRSA: methicillin-resistant Staphylococcus aureus
NPV: negative predictive value
PPV: positive predictive value
ROC: receiver operating characteristic
SD: standard deviation
SOA: service oriented architecture
SOAP: simple object access protocol
UCL: upper control limit
VRE: vancomycin-resistant Enterococcus
XML: extensible markup language
