# Supplementary material for: A Web-Based Multidrug-Resistant Organisms Surveillance and Outbreak Detection System with Rule-Based Classification and Clustering
Source: J Med Internet Res. 2012 Oct 24;14(5):e131. doi: 10.2196/jmir.2056 (PMC3510772; doi:10.2196/jmir.2056)
Supplement: Supplementary file 1 [file jmir_v14i5e131_app1.pdf]

**Appendix 1. Target organisms for surveillance and definition of the multidrug-resistant organisms. The patterns are classified into 5 categories according to classification logics.**

| Pattern | Surveillance target | Antimicrobial disc | Susceptibility testing result |
|---|---|---|---|
| **1.01** | *Staphylococcus aureus* (MRSA) | Oxacillin | I or R[a] |
| **1.02** | *Staphylococcus aureus* (VRSA) | Vancomycin | I or R |
| **1.03** | *Staphylococcus aureus* (TRSA) | Teicoplanin | I or R |
| **1.04** | *Enterococcus faecium* (VRE) | Vancomycin | I or R |
| **1.05** | *Enterococcus fecalis* (VRE) | Vancomycin | I or R |
| **1.06** | Carbapenem-resistant *Escherichia coli* (CREC) | Ertapenem or imipenem or meropenem | I or R |
| **1.07** | Carbapenem-resistant *Klebsiella pneumoniae* (CRKP) | | |
| **1.08** | Carbapenem-resistant *Pseudomonas aeruginosa* (CRPA) | | |
| **1.09** | Carbapenem-resistant *Acinetobacter baumannii* (CRAB) | | |
| **1.10** | Multidrug-Resistance Tuberculosis | Isoniazid and rifampin | I or R |
| **1.11** | Fluoroquinolone -resistant *Pseudomonas aeruginosa* | Ciprofloxacin or levofloxacin | I or R |
| **1.12** | Fluoroquinolone-resistant *Escherichia coli* | | |
| **1.13** | Coagulase-Negative Staphylococci (MRCNS) | Oxacillin | I or R |
| **1.14** | Coagulase-Negative Staphylococci (VRCNS) | Vancomycin | I or R |
| **1.15** | Coagulase-Negative Staphylococci (TRCNS) | Teicoplanin | I or R |
| **1.16** | *Staphylococcus aureus* total | | |
| **1.17** | *Staphylococcus aureus* (MSSA) | Oxacillin | S |
| **1.18** | *Mycobacterium Tuberculosis* total | | |
| **2.01** | *Escherichia coli* (ESBL) | | |
| **2.02** | *Klebsiella pneumoniae* (ESBL) | | |
| **2.03** | *Klebsiella oxytoca* (ESBL) | | |
| **2.04** | *Proteus mirabilis* (ESBL) | | |
| **2.05** | *Escherichia coli* | ≥ 3 classes | I or R |
| **2.06** | *Klebsiella pneumoniae* | ≥ 3 classes | I or R |
| **2.07** | *Klebsiella oxytoca* | ≥ 3 classes | I or R |
| **2.08** | *Proteus mirabilis* | ≥ 3 classes | I or R |
| **2.09** | Carbapenem-resistant *Enterobacter* species (CR *Enterobacter*) | | I or R |
| **2.10** | Carbapenem-resistant *Proteus* species (CR *Proteus*) | | |
| **2.11** | Carbapenem-resistant *Aeromonas* species (CR *Aeromonas*) | | |
| **2.12** | Carbapenem-resistant *Serratia* species (CR *Serratia* ) | | |
| **2.13** | Carbapenem-resistant *Citrobacter* species (CR *Citrobacter*) | | |
| **2.14** | Carbapenem-resistant *Morganella* species (CR *Morganella* ) | | |
| **2.15** | Carbapenem-resistant *Providencia* species (CR *Providencia*) | | |
| **2.16** | Carbapenem-resistant *Klebsiella* species (CR *Klebsiella*) | | |

| | | | |
|---|---|---|---|
| **2.17** | Carbapenem-resistant *Edwardsiella* species (CR *Edwardsiella*) | | |
| **2.18** | Carbapenem-resistant *Ewingella* species (CR *Ewingella*) | | |
| **2.19** | Carbapenem-resistant *Leclercia* species (CR *Leclercia*) | | |
| **2.20** | Carbapenem-resistant *Pantoea* species (CR *Pantoea*) | | |
| **2.21** | Carbapenem-resistant *Salmonella* species (CR *Salmonella*) | | |
| **2.22** | Carbapenem-resistant *Shigella* species (CR *Salmonella*) | | |
| **2.23** | Carbapenem-resistant others | | |
| **3.01** | *Acinetobacter baumannii* (MDRAB) | ≥ 3 classes | I or R |
| **3.02** | *Acinetobacter baumannii* (XDRAB) | ≥ 5 classes | I or R |
| **3.03** | *Pseudomonas aeruginosa* (MDRPA) | ≥ 3 classes | I or R |
| **3.04** | *Pseudomonas aeruginosa* (XDRPA) | ≥ 5 classes | I or R |
| **3.05** | MDR *Acinetobacter* species | ≥ 3 classes | I or R |
| **3.06** | XDR *Acinetobacter* species | ≥ 5 classes | I or R |
| **3.07** | MDR *Pseudomonas* species | ≥ 3 classes | I or R |
| **3.08** | XDR *Pseudomonas* species | ≥ 5 classes | I or R |
| **3.09** | Carbapenem-resistant *Stenotrophomonas maltophilia* (CRSM) | Imipenem or meropenem | I or R |
| **3.10** | Carbapenem-resistant NFGNB (CR NFGNB) | | |
| **3.11** | *Mycobacterium abscessus* | | |
| **3.12** | *Mycobacterium chelonae* | | |
| **4.01** | *Stenotrophomonas maltophilia* | | |
| **4.02** | *Burkholderia cepacia* | | |
| **4.03** | *Chryseobacterium meningosepticum* | | |
| **4.04** | *Chryseobacterium indologenes* | | |
| **4.05** | *Ralstonia pickettii* | | |
| **4.06** | *Clostridium difficile* | | |
| **5.01** | *Candida albicans* | | |
| **5.02** | *Candida tropicalis* | | |
| **5.03** | *Candida glabrata* | | |
| **5.04** | *Candida parapsilosis* | | |
| **5.05** | *Candida krusei* | | |
| **5.06** | *Aspergillus* species total | | |
| **5.07** | *Aspergillus fumigatus* | | |
| **5.08** | *Aspergillus flavus* | | |
| **5.09** | *Aspergillus terreus* | | |
| **5.10** | *Fusarium* species total | | |

[a] Intermediate or resistant.
