# Supplementary material for: A Web-Based Multidrug-Resistant Organisms Surveillance and Outbreak Detection System with Rule-Based Classification and Clustering
Source: J Med Internet Res. 2012 Oct 24;14(5):e131. doi: 10.2196/jmir.2056 (PMC3510772; doi:10.2196/jmir.2056)
Supplement: Supplementary file 2 [file jmir_v14i5e131_app2.pdf]

Real-time Automated MDRO Surveillance System

Jung-Hsuan Wu[1,3], Yee-Chun Chen[4,7], Sung-Huai Hsieh[1,2], Hui-Chi Lin[4], Ying-Yu Chen[1,8], Po-Hsun Cheng[6], Farn Wang[3], Feipei Lai[1,2,3,5]

[1]Information Systems Office, National Taiwan University Hospital, Taipei, Taiwan
[2]Department of Computer Science and Information Engineering, National Taiwan University, Taipei, Taiwan
[3]Department of Electrical Engineering, National Taiwan University, Taipei, Taiwan
[4]Center of Infection Control, National Taiwan University Hospital, Taiwan
[5]Graduate Institute of Biomedical Electronics and Bioinformatics, National Taiwan University, Taiwan
[6]Department of Software Engineering, National Kaohsiung Normal University, Taiwan
[7]Department of Internal Medicine, National Taiwan University Hospital and College of Medicine, Taiwan
[8]Graduate Institute of Networking and Multimedia, National Taiwan University, Taipei, Taiwan

Abstract

A web-based hospital-wide automated Multidrug-Resistant Organisms (MDRO) surveillance system was implemented in a 2200-beds medical center in Taiwan. Raw data (text) from clinical microbiology laboratory (external data provided) were evaluated using group mapping techniques to detect specimen reports positive of 28 patterns of defined MDRO. The time trends of the numbers were monitored by the threshold limits generated based on historical data in a defined period.
As per the concept of Service-Oriented Architecture (SOA), we used web services techniques, which are suitable for integrating heterogeneous platforms, protocols, and applications. The sub-components include Data Collection, Data Detection (candidate detector, conflict merger), Data Analysis (germ criterion, patient criterion, patient in each day criterion) and Report Presentation (visualization, notification).
The performance was evaluated by 2 direct indicators (time cost and accuracy of MDRO detection) and 2 indirect indicators (proportion and time lag of contact isolation). The performance based on 3 of the 4 indicators evaluated in the presence of MDRO system is better than those without and achieve statistically different (p value <0.05, t-test). Automatic surveillance saves up to 10% of infection control man power. In conclusion, this system facilitates medical staff for their operations in controlling MDRO, and improves patient safety. This successful experience of developing the MDRO system can be seamless applied to other medical information systems.

Keywords: Resistance, infection control, surveillance, quality improvement, service-oriented architecture

1. Introduction

The advances in medical care and public health have markedly prolonged the human life span and increased in subpopulation vulnerable to infectious diseases. Healthcare-associated infections are associated with higher morbidity/mortality, prolonged hospitalization and extra cost, the field of infection control has gained much attention over the past 30 years in the perspective of patient safety and quality improvement [1-5]. In developed countries 5 to 10% of infections acquired in the hospital occur as part of an epidemic or cluster [6]. The figure is larger for developing countries. However, outbreaks and part of healthcare-associated infections are potentially preventable with good infection control practice [4,6]. Facing the emerging infectious diseases (such as severe acute respiratory syndrome (SARS), highly pathogenic avian influenza H5N1 and swine influenza A (H1N1)) and reemerging infectious diseases (such as tuberculosis), further demonstrated the impact of infection control on occupational health as well as the continuity of the healthcare settings and the whole society [7]. In order to combat the challenges, the real-time comprehensive surveillance coupled with prompt intervention of infectious diseases is a critical part of infection control.

Multidrug-resistant organisms (MDRO) are bacteria and other microorganisms that have developed resistance to multiple classes of antimicrobial drugs recommended in the therapeutic guidelines. Thus, infections due to MDRO are more likely to receive inappropriate initial therapy and are associated with poor outcome [8-10]. A patient who is exposed to persons with MDRO colonization or infection or environments contaminated with MDRO may become infected or become carriers. In addition, himself/herself has the potential to affect others including in-patients, out-patients, medical staff, and households. Thus, patients with MDRO require special infection control precaution, that is, contact isolation to prevent cross transmission [6,9,11].

Traditionally the monitoring of MDRO relies on the infection control persons to manually look up the long lists of laboratory reports and is labor-intensive, inefficient, experience-dependent and error-prone. With the advances in computer technology and data analysis, timely process

and analysis of large amounts of electronic data become feasible, and is well-known for efficiency and consistency. Several computerized systems for infection control surveillance are developed [12,13]. Thus we hypothesize that a computerized automated MDRO surveillance system will facilitate and improve the quality of infection control.

We have developed a web-based MDRO system in a 2200-beds medical center. In external point of view, it follows the concept of service oriented architecture that collects the laboratory reports and feedbacks the detected MDROs to the data provider by web service. In internal point of view, it presents the occurrence of MDRO in the population level and notifies the medical staffs that the patients have MDRO colonization or infection and who need special attention. This system monitors MDRO every day routinely and every hour if indicated in emergent situation. The achievement of this system is evaluated in section 3 and this experience can be seamless applied to other medical-related information systems.

In the following sections, we first elaborate our MDRO system in section 2. In Section 3, the system assessment methods and results are provided. Finally, in section 4 we discuss the strength and weakness of the system and high-light future implication.

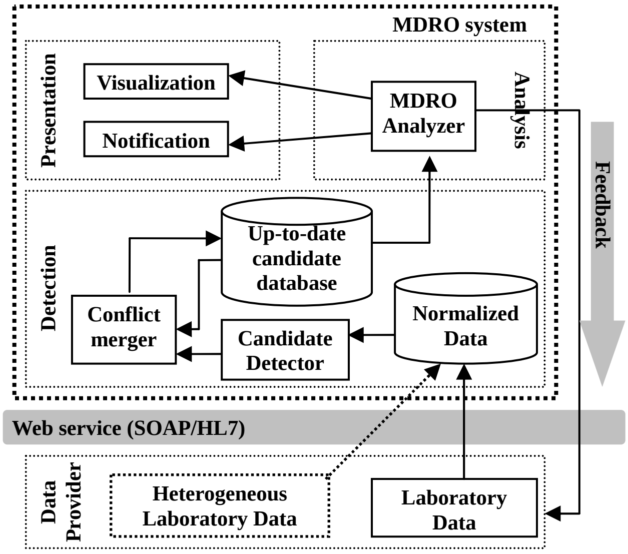

Fig. 1: System architecture of web-based MDRO system

2. Implementation of MDRO system

Figure 1 shows the architecture of the MDRO system. It mainly consists of three parts. First part collects the laboratory raw data (text) by web service from the external data providers (clinical microbiology laboratory). The normalized data will be kept in the internal database. The second part detects the MDROs and keeps them in the up-to-date candidate database. By analyzing these detected MDROs in the third part, we can not only assist the surveillance of MDRO but also feedback to the data provider for their further cautions. The last part presents the analyzed results. The communication between MDRO system and the external data provider is via web service and the data format follows the HL7 [14]. This service oriented architecture can easily provide the MDRO service to other heterogeneous information systems. In the following subsections, we will introduce these components in details.

2.1 MDRO Detection

Candidate Detector According to the local epidemiology and literature review [8-11,15], we re-organized a total of 28 MDRO patterns (organisms and their antibiograms). Any specimen report from the data providers that matches one of the 28 patterns will be recognized as a candidate MDRO report and saved in the database for further analysis. The 28 MDRO patterns are shown in Table 1.

All clinical specimens registered for culture (for example, bacteria) may have one or more results: negative of bacteria, one bacterium but without antimicrobial susceptibility results, one bacterium and with antimicrobial susceptibility results, or two or more bacteria. For certain bacteria, such as *Escherichia coli*, special enzyme (extended spectrum beta-lactamase (ESBL)) producing strain is identified by special test. The final report is *Escherichia coli* (ESBL). Classification of antimicrobial susceptibility results is based on disc diffusion method performed in clinical microbiology laboratory. Multidrug-resistant non-fermentative gram-negative bacteria such as *Acinetobacter baumannii* (MDRAB) and *Pseudomonas aeruginosa* (MDRPA) were defined as those resistant to three or more classes of antimicrobial agents among the 7 classes evaluated (Appendix 1). Extensively drug-resistant *A. baumannii* (XDRAB) was defined as those resistant to five or more classes of antimicrobial agents among the 7 classes evaluated.

Table 1. Types of MDRO patterns and their definition

Pattern No	Surveillance target	Antimicrobial disc	Reaction
1.01	*Staphylococcus aureus* (MRSA)	Oxacillin	R or I
1.02	*Staphylococcus aureus* (VRSA)	Vancomycin	R or I
1.03	*Staphylococcus aureus* (TRSA)	Teicoplanin	R or I
1.04	*Enterococcus faecium* (VRE)	Vancomycin	R or I
1.05	*Enterococcus fecalis* (VRE)	Vancomycin	R or I
1.06	Carbapenem-resistant *Escherichia coli* (CREC)	Ertapenem or Imipenem or Meropenem	R or I
1.07	Carbapenem-resistant *Klebsiella pneumoniae* (CRKP)	Ertapenem or Imipenem or Meropenem	R or I
1.08	Carbapenem-resistant *Pseudomonas aeruginosa* (CRPA)	Imipenem or Meropenem	R or I
1.09	Carbapenem-resistant *Acinetobacter baumannii* (CRAB)	Imipenem or Meropenem	R or I
1.10	Multidrug-resistance *Mycobacterium tuberculosis*	Isoniazid and Rifampin	R or I
1.11	Fluoroquinolone -resistant *Pseudomonas aeruginosa*	Ciprofloxacin or Levofloxacin	R or I
1.12	Fluoroquinolone-resistant *Escherichia coli*	Ciprofloxacin or Levofloxacin	R or I

1.13	Fluoroquinolone-resistant *Escherichia coli*	Ciprofloxacin or Levofloxacin	R or I
1.14	Methicillin-resistant coagulase-negative staphylococci (MRCNS)	Oxacillin	R or I
1.15	Vancomycin -resistant coagulase-negative staphylococci (VRCNS)	Vancomycin	R or I
1.16	Teicoplanin -resistant coagulase-negative staphylococci (TRCNS)	Teicoplanin	R or I
2.01	*Escherichia coli* (ESBL)		
2.02	*Klebsiella pneumoniae* (ESBL)		
2.03	*Klebsiella oxytoca* (ESBL)		
2.04	*Proteus mirabilis* (ESBL)		
3.01	*Acinetobacter baumannii* (MDRAB)	≧ 3 classes	R or I
3.02	*Acinetobacter baumannii* (XDRAB)	≧ 5 classes	R or I
3.03	*Pseudomonas aeruginosa* (MDRPA)	≧ 3 classes	R or I
3.04	*Pseudomonas aeruginosa* (XDRPA)	≧ 5 classes	R or I
4.01	*Stenotrophomonas maltophilia*		
4.02	*Burkholderia cepacia*		
4.03	*Chryseobacterium meningosepticum*		
4.04	*Chryseobacterium indologenes*		

As shown in Table 1, the patterns can be further classified into 4 categories. Each category has different detection mechanism. To show the detection mechanism of each category, we explain several patterns as follows.

Pattern 1.01. If the organism "*Staphylococcus aureus*" is found in the culture of a specimen and the antibiotic test result is resistant (R) or intermediate (I), then this specimen matches the pattern 1.01 which is a candidate specimen report as "*Staphylococcus aureus* (MRSA)".

Pattern 3.01. If the organism "*Acinetobacter baumannii*" is found in the culture of a specimen and the antibiotic test results are resistant (R) or intermediate (I) to 3 or more classes of antibiotic disc, then this specimen matches the pattern 3.01 which is a candidate specimen report of the "*MDRAB*" infection.

Pattern 4.01. Since categories 2 and 3 have the same detection mechanism, we take the pattern 4.01 as an example. *Stenotrophomonas maltophilia* is almost associated with multiple drug resistance. If the organism "*Stenotrophomonas maltophilia*" is found in the culture of a specimen, then this specimen matches the pattern 4.01 which is a candidate specimen report as "*Stenotrophomonas maltophilia*".

Conflict Merger Since the MDRO system is running for the real-time purpose, it will suffer from the problems of conflicts among no report, preliminary data and final report for an indicated specimen. The cultured organisms and the antibiotic test results vary over the time of culture. Thus a specimen report negative of MDRO may become positive at next moment. An MDRO specimen report can be negative or match another MDRO pattern at next moment if a second organism exists. To solve the conflicts, a mechanism is developed and explained as follows.

Given a time period ***P***, let ***A*** be the set of specimen reports in up-to-date candidate database which are registered for culture inside period ***P***. Let ***B*** be the set of specimen reports from data providers which are registered for culture inside period ***P*** and match certain MDRO patterns. The 3 rules to solve the conflicts are:

1. For each specimen report in ($\boldsymbol{A} \cap \boldsymbol{B}$), if the cultured results in ***A*** do not match them in ***B***, then update ***A*** with the results in ***B***.
2. For each specimen report in (***A***-***B***), we mark it as not MDRO.
3. For each specimen report in (***B***-***A***), we add it into the up-to-date candidate database.

Since August 2008, the MDRO system runs every day routinely in this hospital to detect the candidate specimen reports which were generated in the past 7 days. The running frequency and detecting interval can be increased and extended to handle more organisms of concern in an emergent situation such as during epidemic or outbreak of infection.

Claim: The MDRO system detects MDRO specimen reports with 100% accuracy.

We will show this claim in two parts.

1. *The candidate detector detects all MDRO specimen reports.*
   During the established step, we check the accuracy of detection results using independent methods and revise the program till 100% accuracy. More efforts are for data cleaning and clarification of definition of MDRO.
2. *The conflict merger guarantees the candidate specimen reports are up-to-date*
   Consider the candidate detector runs in two different times. Then there are 4 cases to analyze.
   - If a specimen report is MDRO in the previous time and the latest time, then rule 1 guarantees it is up-to-date. Note that if the matched patterns differ in these two times, the medical staff will be informed to adjust the intervention (infection control precaution).
   - If a specimen report is MDRO in the previous time and not MDRO in the latest time, then rule 2 guarantees it is up-to-date. The medical staff will also be informed in this situation to adjust the intervention.
   - If a specimen report is not MDRO in the previous time and MDRO in the latest time, then rule 3 guarantees it is up-to-date. In this case, the medical staff will be informed to take relevant intervention.
   - If a specimen report is not MDRO in the previous time and the latest time, then it is still up-to-date.

With the above two points, the claim follows.

2.2 MDRO Analysis

In this subsection, we will introduce the three criteria that we use to monitor the occurrence of MDRO in a ward, a department or the hospital. Given a MDRO pattern, the three criteria *germ criterion*, *patient criterion*, and the

patient in each day criterion are counted. Note that each specimen can have one or more reports, and each report can match one or more MDRO patterns. For example, a XDRAB specimen report matches both XDRAB and MDRAB patterns.

Germ Criterion With this criterion, we count the numbers of MDRO specimen reports in a given period. One patient may have one or more specimens (from same body sites or different body sites) positive of MDRO during the specific time period.

Patient Criterion With this criterion, we count the numbers of patients who have MDRO specimen reports in a given period. We also distinguish a patient who is newly colonized or infected with MDRO if he/she did not have MDRO specimen reports in the past 3 months before release of the current MDRO specimen report.

Patient in Each Day Criterion With this criterion, we count the numbers of patients who have MDRO specimen reports in a given period. Note that each patient is counted at most 1 in each day.

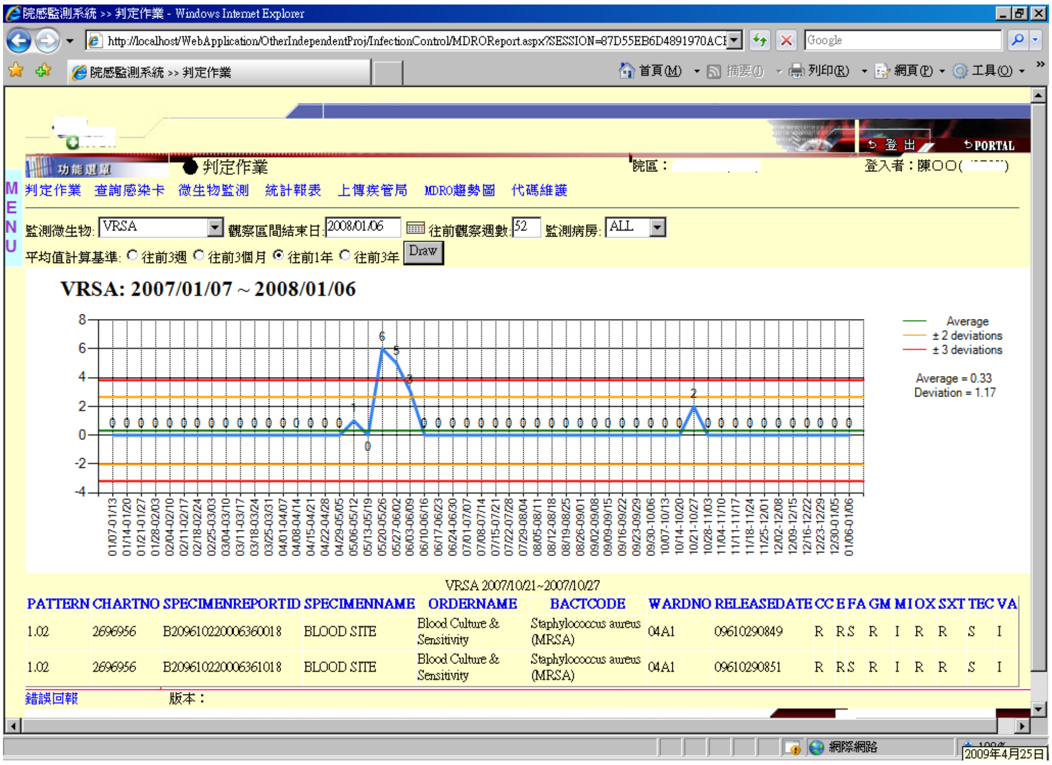

Fig. 2: the trend of infection spread and the details of each number

2.3 MDRO Presentation

We have adopted several IT techniques to assist the surveillance of MDRO. These techniques include email, web, Ajax and MSChart [16]. In the following, we show how these techniques are used.

Visualization

To visualize the analyzed MDRO specimen report, we utilize the web, Ajax and MSChart to draw the time trends of the three criteria mentioned in subsection 3.2 to describe the occurrence of MDRO in the population level. Figure 3 demonstrates the trend of VRSA (pattern 1.02) with germ criterion. The green central line shows the average number of VRSA in the past defined period (3 months, one year or 3 years). The orange and red lines show the number of 2 and 3 standard deviations above and below the central lines, respectively. With these control lines (threshold limits), the infection control personnel or other medical staff involved in control of MDRO may judge if a serious outbreak of MDRO is happening or not.

Besides the trend of numbers, the medical staff can also retrieve the detailed information of specimen reports contributing to a certain number (the lower part of Figure 3). With the two-level embedded function, they can rapidly response to control further spread of MDRO.

Notification

Besides the visualization, the medical staff who in charge of the primary care of the patient with MDRO specimen reports will also be informed every day by e-mail. For every MDRO patterns, the medical staff are informed not only the newly occurrence of MDRO specimen reports (to initiate special infection control precaution), but also the specimen reports which are previously detected as MDRO but no longer MDRO lately (to discontinue special infection control precaution). With this notification, the medical staff can save their time of searching for lab report and can response timely when a MDRO report is released.

3. System Assessments

To assess the MDRO system, we define 2 direct indicators and 2 indirect indicators to evaluate its performance. The direct indicators include time cost of MDRO and accuracy of MDRO. The indirect indicators include proportion and time lag of contact isolation.

For direct indicators, 10 hospital staff were invited to evaluate the performance. These included 8 infection control nurses with an average of 5.5 years' experience. Two hospital staff did not have any experience in infection control and were taught for one hour to identify MDRO based on definition in order to perform the test. Each

hospital staff was assigned 4 sets of 100 randomly selected clinical isolates to evaluate the performance.

As the MDRO system was launched to facilitate infection control program in population level since Aug. 2008, we compared the indirect indicators in the absence of MDRO system (April 1, 2008 ~July 9, 2008) and in the presence of MDRO system (Sept. 1, 2008 ~ Dec. 9, 2008).

Table 2. Assessment of MDRO system performance

	Without MDRO System		With MDRO System		
	Mean	S.D	Mean	S.D	P value*
Time cost of MDRO detection per day (mins)	450	192.19	0	0	<.0001
Accuracy of MDRO detection (%)	63.889	26.414	100	0	<.0001
Proportion of contact isolation (%)	16.525	16.466	25.447	22.126	0.0014
Time lag of contact isolation (hours)	307.81	581.88	297.42	487.12	0.8912

Time cost of MDRO detection

The time cost of MDRO detection in the absence of MDRO system was estimated by the hospital staff person-hour to identify the MDRO in 100 isolates multiplied by numbers of total clinical isolates per day divided by 100. If we assume that the MDRO system cost zero time to detect MDRO, thus, the cost saving could be estimated further according to the salary of infection control persons in each country.

Accuracy of MDRO detection

The accuracy was defined by the proportion of MDRO specimen reports identified:

$$\frac{\text{Number of MDRO specimen reports identified}}{\text{Number of MDRO specimen reports}}$$

Note that according to our previous claim, the accuracy of MDRO with the MDRO system in any given data set was 100%.

Proportion of contact isolation

According to isolation precaution guideline at this hospital, patients with MDRO colonization/infection should receive contact isolation. The proportion of contact isolation is defined by the number of contact isolation orders divided by the number of patients with MDRO specimen reports. That is,

$$\frac{\text{Number of patients with contact isolation orders}}{\text{Number of patients with MDRO specimen reports}}$$

Time lag of contact isolation

Time lag of contact isolation is defined by the time interval between generation of final report of MDRO and the generation of contact isolation order.

The results are shown in Table 2. Without MDRO system, it took around 8 person-hours to detect these MDRO per day in this 2200-bed medical center. Besides, the accuracy of MDRO detection was poor with an average of 63%. The performance based on 3 of the 4 indicators evaluated in the presence of MDRO system is better than those without and achieve statistically different (p value <0.05, t-test). With the assist of the MDRO system, infection control nurses were more available to inform primary care physicians or nurses in the wards to isolate the patients with MDRO and conduct audit for compliance of infection control precaution on the floor. As shown in Table 3, proportion of contact isolation increased.

4. Conclusion and Discussion

This web-based real-time automated comprehensive MDRO surveillance system can detect and monitor 28 MDRO of concern based on raw data generated from external data provider with excellent and consistent quality. If the monitoring activities run on a daily basis, it saves 8 person-hours which are equal to 10% of infection control man power. Human performance varies by experience and is error prone. It is of great concern in healthcare system in the perspective of patient safety. Furthermore, if the pathogen surveyed is associated with high transmissibility, morbidity and mortality, failure to detect and then to initiate appropriate infection control precaution is more likely to have tremendous adverse impact for the healthcare settings. The weakness of current infection control surveillance system is obvious. The cost benefit of the MDRO system warranted further explored.

Despite of the introduction of MDRO system, the time lag between generation of MDRO report and performance of contact isolation did not improve. The main reasons for time lag included time lag for infection control nurses to inform primary care physicians or nurses and the latter to perform isolation precaution. Therefore, in the future version, we will include alert signal directly in medical information system (PORTAL) [17].

In conclusion, we have developed a web-based hospital-wide MDRO surveillance system. This system can facilitate entire medical staff for their operations in the hospital, and improve patient safety as well as the quality of medical cares. This successful experience of developing MDRO system can be easily applied to other related system. Meanwhile, the SOA architecture of the system can also be seamless integrated to HIS system.

Acknowledgments

The project was in part supported by grants (CDC97-DC-1005 and DOH 98-DC-1007) from the Center for Disease Control, Department of Health, Taiwan.

5. References

[1] Burke JP. *Infection Control - A problem for patient safety.* N Engl J Med 2003;348:651.

[2] Allegranzi B, Storr J, Dziekan G, et al. *The first Global Patient Safety Challenge "Clean Care is Safer Care": from launch to current progress and achievements.* J Hosp Infect 2007;65:115-23.

[3] Sheng WH, Wang JT, Lu DCT, et al. *Comparative impact of hospital-acquired infections on medical costs, length of hospital stay and outcome between community hospitals and medical centers.* J Hosp Infect 2005;59:205-14.

[4] Grundmann H, Barwolff S, Tami A, et al. *How many*

infections are caused by patient-to-patient transmission intensive care units? Crit Care Med 2005;33:946-51.

[5] Lindenauer PK, Remus D, Roman S, et al. *Public reporting and pay for performance in hospital quality improvement.* New Engl J Med 2007;356:486-96.

[6] Wenzel RP, Bearman GML, Brewer TF, et al. *A Guide to Infection Control in the Hospital, 4th edition.* International Society of Infectious Diseases, 2008.

[7] Chen YC, Huang LM, Chan CC, et al. *SARS in hospital emergency room.* Emerg Infect Dis 2004;10:782-8.

[8] Nicolle LE. *Infection Control Programmes to Control Antimicrobial Resistance*. World Health Organization. 2001.

[9] Muto CA, Jernigan JA, Ostrowsky BE, et al. *SHEA Guideline for preventing nosocomial transmission of multidrug-resistant strains of Staphylococcus aureus and Enterococcus*. Infect Control Hospital Epidemiol 2003;5:363-386.

[10] CDC. *Recommendations for preventing the spread of vancomycin resistance recommendations of the hospital infection control practices advisory committee (HICPAC).* MMWR 1995；44：1-13.

[11] *Guidelines for the management of patients with vancomycin-resistant enterococci (VRE) colonisation/ Infection. Part 3: Guidelines for the management of patients in long-term care facilities (LTCF) and rehabilitation units*. Human Services 1999；11-14.

[12] Kho AN, Dexter PR, Warvel JS, et al. *An effective computerized reminder for contact isolation of patients colonized or infected with resistant organisms*. International Journal of Medical Informatics, Vol. 77, No. 3. (March 2008), pp. 194-198.

[13] Pittet D, Safran E, Harbarth S, et al. *Automatic alerts for methicillin-resistant Staphylococcus aureus surveillance and control: role of a hospital information system.* Infection Control and Hospital Epidemiology, 1996;17(8):496-502.

[14] Health Level Seven (HL7). HL7 Standard v2.5, 2003, chapter4 p4-14 - p4-17.

[15] CDC. *Nosocomial enterococci resistant to vancomycin-United States, 1989-1993*. MMWR 1993;42:597-599.

[16] *Microsoft Chart Controls.* http://code.msdn.microsoft.com/mschart

[17] Hsieh SH, Hsieh, S.L, Weng, Y.C, et al. *Middleware based Inpatient Healthcare Information System.* Bioinformatics and Bioengineering, 2007. BIBE 2007.

[18] Kucher N, Koo S, Quiroz R, et al. *Electronic alerts to prevent venous thromboembolism among hospitalized patients.* N. Engl. J. Med. 352 (2005) 969–977.

A Appendix

A.1 Classification of antibiotic susceptibility results for non-fermentative gram-negative bacteria.

Class	Antibiotic disc
Aminoglycoisde	gentamicin
	amikacin
Anti-pseudomonas fluoroquinolone	ciprofloxacin
	levofloxacin
Anti-pseudomonas cephalosporine	cefepime
	ceftazidime
Extended spectrum penicillin	ticarcillin-clavulanic acid
	piperacillin-tazobactam
Anti-pseudomonas carbapenem	meropenem
	imipenem
	ertapenem
sulbactam	sulbactam
Polymyxin	colistin
