# Supplementary material for: A Web-Based Multidrug-Resistant Organisms Surveillance and Outbreak Detection System with Rule-Based Classification and Clustering
Source: J Med Internet Res. 2012 Oct 24;14(5):e131. doi: 10.2196/jmir.2056 (PMC3510772; doi:10.2196/jmir.2056)
Supplement: Supplementary file 3 [file jmir_v14i5e131_app3.pdf]

**Appendix 3- Performance in outbreak detection according to a variety of germ criterion and upper control limits (UCL), with and without clustering analysis**

| | UCL | Sensitivity[a] | Specificity[b] | PPV[c] | NPV[d] | AUC (95% CI)[e] |
|---|---|---|---|---|---|---|
| Without clustering | 99% CI[f] | 63.3 (19/30) | 89.7 (673/750) | 19.8 (19/96) | 98.4 (673/684) | 0.765 (0.661-0.870) |
| | 95% CI | 76.7 (23/30) | 87.6 (657/750) | 19.8 (23/116) | 98.9 (657/664) | 0.821 (0.732-0.911) |
| | 90% CI | 90.0 (27/30) | 84.0 (630/750) | 18.4 (27/147) | 99.5 (630/633) | 0.870 (0.806-0.934) |
| | 85%CI | 90.0 (27/30) | 82.7 (620/750) | 17.2 (27/157) | 99.5 (620/623) | 0.863 (0.799-0.928) |
| | 3SD[h] | 33.3 (10/30) | 90.8 (681/750) | 12.7 (10/79) | 97.1 (681/701) | 0.621 (0.505-0.736) |
| | 2SD | 53.3 (16/30) | 89.3 (670/750) | 16.7 (16/96) | 98.0 (670/684) | 0.713 (0.603-0.824) |
| | 1SD | 80.0 (24/30) | 84.4 (633/750) | 17.0 (24/141) | 99.1 (633/639) | 0.822 (0.783-0.906) |
| With clustering (d=0)[g] | 99% CI | 80.0 (24/30) | 90.1 (676/750) | 24.5 (24/98) | 99.1 (676/682) | 0.851 (0.766-0.935) |
| | 95% CI | 93.3 (28/30) | 88.1 (661/750) | 23.9 (28/117) | 99.7 (661/663) | 0.907 (0.854-0.961) |
| | 90% CI | 100 (30/30) | 86.7 (650/750) | 23.1 (30/130) | 100 (650/650) | 0.933 (0.913-0.953) |
| | 85%CI | 100 (30/30) | 86.0 (645/750) | 22.2 (30/135) | 100 (645/645) | 0.930 (0.909-0.951) |
| | 3SD | 70.0 (21/30) | 90.7 (680/750) | 23.1 (21/91) | 98.7 (680/689) | 0.803 (0.705-0.901) |
| | 2SD | 83.3 (25/30) | 89.6 (672/750) | 24.3 (25/103) | 99.3 (672/677) | 0.865 (0.786-0.943) |
| | 1SD | 96.7 (29/30) | 87.3 (655/750) | 23.4 (29/124) | 99.8 (655/656) | 0.920 (0.879-0.961) |

[a] Sensitivity =TP/ (TP+FN), [b] Specificity =TN/ (TN+FP), [c] Positive predictive value (PPV)= TP/ (TP+FP), [d] Negative predictive value (NPV)= TN/ (TN+FN), [e] AUC: Area under receiver operating characteristic curve, [f] CI: confidence interval, [g] d: cutting Euclidean distance. True positive (TP): An

outbreak correctly identified as an outbreak. False positive (FP): A non-outbreak wrongly identified as an outbreak. True negative (TN): A non-outbreak correctly identified as a non-outbreak. False negative (FN): An outbreak wrongly identified as a non-outbreak.
