# Supplementary material for: A Web-Based Multidrug-Resistant Organisms Surveillance and Outbreak Detection System with Rule-Based Classification and Clustering
Source: J Med Internet Res. 2012 Oct 24;14(5):e131. doi: 10.2196/jmir.2056 (PMC3510772; doi:10.2196/jmir.2056)
Supplement: Supplementary file 4 [file jmir_v14i5e131_app4.pdf]

Appendix 4- Performance in outbreak detection according to patient criterion and a variety of upper control limits (UCL), with and without clustering analysis

	UCL	Sensitivity[a]	Specificity[b]	PPV[c]	NPV[d]	AUC (95% CI)[e]
Without clustering	99% CI [f]	46.7 (14/30)	90.3 (677/750)	16.1 (14/87)	97.7 (677/693)	0.685 (0.571-0.798)
	95% CI	76.7 (23/30)	87.5 (656/750)	19.7 (23/117)	98.9 (656/663)	0.821 (0.931-0.910)
	90% CI	86.7 (26/30)	82.8 (621/750)	16.8 (26/155)	99.4 (621/625)	0.847 (0.775-0.919)
	85%CI	86.7 (26/30)	81.1 (608/750)	15.5 (26/168)	99.3 (608/612)	0.839 (0.766-0.911)
	3SD [h]	30.0 (9/30)	90.9 (682/750)	11.7 (9/77)	97.0 (682/703)	0.605 (0.490-0.720)
	2SD	50.0 (15/30)	89.1 (668/750)	15.5 (15/97)	97.8 (668/683)	0.695 (0.583-0.807)
	1SD	86.7 (26/30)	83.5 (626/750)	17.3 (26/150)	99.4 (626/630)	0.851 (0.779-0.923)
With clustering (d=1)[g]	99% CI	46.7 (14/30)	90.3 (677/750)	16.1 (14/87)	97.7 (677/693)	0.685 (0.571-0.798)
	95% CI	76.7 (23/30)	87.5 (656/750)	19.7 (23/117)	98.9 (656/663)	0.821 (0.731-0.910)
	90% CI	86.7 (26/30)	82.7 (620/750)	16.7 (26/156)	99.4 (620/624)	0.847 (0.775-0.919)
	85%CI	86.7 (26/30)	80.9 (607/750)	15.4 (26/169)	99.3 (607/611)	0.838 (0.766-0.910)
	3SD	30.0 (9/30)	90.9 (682/750)	11.7 (9/77)	97.0 (682/703)	0.605 (0.490-0.720)
	2SD	50.0 (15/30)	89.1 (668/750)	15.5 (15/97)	97.8 (668/683)	0.695 (0.583-0.807)
	1SD	86.7 (26/30)	83.2 (624/750)	17.1 (26/152)	99.4 (624/628)	0.849 (0.777-0.921)
With clustering (d=0)	99% CI	66.7 (20/30)	90.0 (675/750)	21.1 (20/95)	98.5 (675/685)	0.783 (0.682-0.885)
	95% CI	83.3 (25/30)	87.6 (657/750)	21.2 (25/118)	99.2 (657/662)	0.855 (0.776-0.933)
	90% CI	83.3 (25/30)	84.5 (634/750)	17.7 (25/141)	99.2 (634/639)	0.839 (0.761-0.918)
	85%CI	90.0 (27/30)	83.6 (627/750)	18.0 (27/150)	99.5 (627/630)	0.868 (0.804-0.932)
	3SD	60.0 (18/30)	90.5 (679/750)	20.2 (18/89)	98.3 (679/691)	0.753 (0.646-0.860)
	2SD	76.7 (23/30)	89.5 (671/750)	22.5 (23/102)	99.0 (671/678)	0.831 (0.741-0.920)

	1SD	86.7 (26/30)	85.5 (641/750)	19.3 (26/135)	99.4 (641/645)	0.861 (0.789-0.932)

[a] Sensitivity =TP/ (TP+FN), [b] Specificity =TN/ (TN+FP), [c] Positive predictive value (PPV)= TP/ (TP+FP), [d] Negative predictive value (NPV)= TN/ (TN+FN), [e] AUC: Area under receiver operating characteristic curve, [f] CI: confidence interval, [g] d: cutting Euclidean distance. True positive (TP): An outbreak correctly identified as an outbreak. False positive (FP): A non-outbreak wrongly identified as an outbreak. True negative (TN): A non-outbreak correctly identified as a non-outbreak. False negative (FN): An outbreak wrongly identified as a non-outbreak.
