# Supplementary material for: A Web-Based Multidrug-Resistant Organisms Surveillance and Outbreak Detection System with Rule-Based Classification and Clustering
Source: J Med Internet Res. 2012 Oct 24;14(5):e131. doi: 10.2196/jmir.2056 (PMC3510772; doi:10.2196/jmir.2056)
Supplement: Supplementary file 5 [file jmir_v14i5e131_app5.pdf]

Appendix 5- Performance in outbreak detection according to incident patient criterion and a variety of upper control limit (UCL), with and without clustering analysis

	UCL	Sensitivity[a]	Specificity[b]	PPV[c]	NPV[d]	AUC (95% CI)[e]
Without clustering	99% CI [f]	53.3 (16/30)	90.5 (679/750)	18.4 (16/87)	98.0 (679/693)	0.719 (0.608-0.830)
	95% CI	60.0 (18/30)	83.9 (629/750)	12.9 (18/139)	98.1 (629/641)	0.736 (0.630-0.842)
	90% CI	76.7 (23/30)	83.9 (629/750)	16.0 (23/144)	99.0 (629/636)	0.803 (0.714-0.892)
	85% CI	76.7 (23/30)	83.2 (624/750)	15.4 (23/149)	98.9 (624/631)	0.799 (0.710-0.888)
	3SD [h]	46.7 (14/30)	90.8 (681/750)	16.9 (14/83)	97.7 (681/697)	0.687 (0.574-0.801)
	2SD	60.0 (18/30)	87.7 (658/750)	16.4 (18/110)	98.2 (658/670)	0.739 (0.632-0.845)
	1SD	73.3 (22/30)	83.6 (627/750)	15.2 (22/145)	98.7 (627/635)	0.785 (0.691-0.878)
With clustering (d=1)[g]	99% CI	46.7 (14/30)	90.1 (676/750)	15.9 (14/88)	97.7 (676/692)	0.717 (0.606-0.828)
	95% CI	60.0 (18/30)	87.1 (653/750)	15.7 (18/115)	98.2 (653/665)	0.717 (0.606-0.828)
	90% CI	76.7 (23/30)	83.9 (629/750)	16.0 (23/144)	98.9 (629/636)	0.735 (0.629-0.841)
	85%CI	76.7 (23/30)	83.2 (624/750)	15.4 (23/149)	98.9 (624/631)	0.799 (0.710-0.888)
	3SD	46.7 (14/30)	90.5 (679/750)	16.5 (14/85)	97.7 (679/695)	0.803 (0.714-0.892)
	2SD	60.0 (18/30)	87.6 (657/750)	16.2 (18/111)	98.2 (657/669)	0.738 (0.632-0.844)
	1SD	73.3 (22/30)	83.6 (627/750)	15.2 (22/145)	98.7 (627/635)	0.785 (0.691-0.878)
With clustering (d=0)	99% CI	70.0 (21/30)	90.4 (678/750)	22.6 (21/93)	98.7 (678/687)	0.802 (0.704-0.900)
	95% CI	76.7 (23/30)	88.3 (662/750)	20.7 (23/111)	99.0 (662/669)	0.825 (0.735-0.914)
	90% CI	80.0 (24/30)	87.2 (654/750)	20.0 (24/120)	99.0 (654/660)	0.836 (0.752-0.920)
	85% CI	80.0 (24/30)	87.2 (654/750)	20.0 (24/120)	99.1 (654/660)	0.836 (0.752-0.920)
	3SD	70.0 (21/30)	90.4 (678/750)	22.6 (21/93)	98.7 (678/687)	0.802 (0.704-0.900)
	2SD	76.7 (23/30)	88.8	21.5	99.0	0.827 (0.738-

			(666/750)	(23/107)	(666/673)	0.917)
	1SD	80.0 (24/30)	87.3 (655/750)	20.2 (24/119)	99.1 (655/661)	0.837 (0.752-0.921)

[a] Sensitivity =TP/ (TP+FN), [b] Specificity =TN/ (TN+FP), [c] Positive predictive value (PPV)= TP/ (TP+FP), [d] Negative predictive value (NPV)= TN/ (TN+FN), [e] AUC: Area under receiver operating characteristic curve, [f] CI: confidence interval, [g] d: cutting Euclidean distance. True positive (TP): An outbreak correctly identified as an outbreak. False positive (FP): A non-outbreak wrongly identified as an outbreak. True negative (TN): A non-outbreak correctly identified as a non-outbreak. False negative (FN): An outbreak wrongly identified as a non-outbreak.
